# Genome-wide methylation patterns provide insight into differences in breast tumor biology between American women of African and European ancestry

**DOI:** 10.18632/oncotarget.1599

**Published:** 2013-11-29

**Authors:** Christine B. Ambrosone, Allyson C. Young, Lara E. Sucheston, Dan Wang, Yan Li, Song Liu, Li Tang, Quang Hu, Jo L. Freudenheim, Peter G. Shields, Carl D. Morrison, Kitaw Demissie, Michael J. Higgins

**Affiliations:** ^1^ Department of Cancer Prevention and Control, Roswell Park Cancer Institute, Buffalo, NY; ^2^ Department of Molecular and Cell Biology, Roswell Park Cancer Institute, Buffalo, NY; ^3^ Department of Biostatistics and Bioinformatics, Roswell Park Cancer Institute, Buffalo, NY; ^4^ Department of Social and Preventive Medicine, University at Buffalo, Buffalo, NY; ^5^ College of Medicine, Ohio State University Comprehensive Cancer Center, Columbus, OH; ^6^ Department of Pathology, Roswell Park Cancer Institute, Buffalo, NY; ^7^ Department of Epidemiology, Rutgers School of Public Health and Cancer Institute of New Jersey, NJ

**Keywords:** DNA methylation, breast cancer, disparities, estrogen receptor, African-American, genome-wide

## Abstract

American women of African ancestry (AA) are more likely than European-Americans (EA) to be diagnosed with aggressive, estrogen receptor (ER) negative breast tumors; mechanisms underlying these disparities are poorly understood. We conducted a genome wide (450K loci) methylation analysis to determine if there were differences in DNA methylation patterns between tumors from AA and EA women and if these differences were similar for both ER positive and ER negative breast cancer. Methylation levels at CpG loci within CpG islands (CGI)s and CGI-shores were significantly higher in tumors (n=138) than in reduction mammoplasty samples (n=124). In hierarchical cluster analysis, there was separation between tumor and normal samples, and in tumors, there was delineation by ER status, but not by ancestry. However, differential methylation analysis identified 157 CpG loci with a mean β value difference of at least 0.17 between races, with almost twice as many differences in ER-negative tumors compared to ER-positive cancers. This first genome-wide methylation study to address disparities indicates that there are likely differing etiologic pathways for the development of ER negative breast cancer between AA and EA women. Further investigation of the genes most differentially methylated by race in ER negative tumors can guide new approaches for cancer prevention and targeted therapies, and elucidate the biologic basis of breast cancer disparities.

## INTRODUCTION

Although American women of European ancestry (EA) overall, have higher breast cancer incidence than American women with African ancestry (AA), AA women are more likely to have aggressive tumors, characterized by higher grade, higher proliferative indices, lack of expression of estrogen receptor (ER) and progesterone receptor (PR), and the absence of HER-2 amplification [1]. These ‘triple negative’ breast cancers are most lethal because of their unresponsiveness to hormonal therapy or to Herceptin, making fewer treatment options available. The reasons for these racial differences in breast cancer biology are unknown. While the epidemiology of breast cancer (e.g., age at onset and aggressive characteristics) differs between AA and EA women, it is unclear if tumor biology differs between groups. It may be that breast cancer subtypes are similar in EAs and AAs, but that the risk factors for more aggressive cancers are more prevalent among AA women, or it may be that the biology of the tumors differs between these groups.

Aberrant DNA methylation is a commonly occurring alteration in breast tumors, and there is some evidence that there are differences in methylation associated with different breast cancer risk factors [2,3]. DNA methylation is one mechanism through which genetic and non-genetic factors could affect development of breast cancer, and which could elucidate disparities in aggressiveness. Changes could include either hypomethylation, which may allow for expression of factors that would increase growth potential, or hypermethylation, which could silence genes necessary to prevent more aggressive tumor growth. If tumor biology does indeed differ between AA and EA women, it is also possible that biologic processes, including methylation, would differ by race as well, with mechanistic pathways to aggressive tumors differing between AA and EA women.

There is some evidence for differential methylation patterns between AAs and EAs in normal tissue, including reports from a study of DNA methylation in leukocytes from women in a multi-ethnic New York City Birth Cohort [4], and from analysis of umbilical cord blood from newborns [5]. Racial differences in methylation of 5 genes were also noted in breast tissue from women undergoing reduction mammoplasty [6]. In a study of both normal human prostate tissue and prostate cancer, 6 genes also showed differential methylation between AAs and EAs [7].

For assessing potential racial differences in DNA methylation in breast tumors, Mehrotra and colleagues used methylation-specific PCR to examine genes known to be involved in breast cancer, comparing differential methylation in AA and EA women by ER and PR status, and by age [8]. Among women diagnosed before age 50 and with tumors that were ER-/PR-, AA women had a significantly higher frequency of methylation in 4 of 5 genes evaluated: *HIN-1* (79% in AA and 19% in EA), *Twist* (67% and 16%), *Cyclin D2* (64% and 19%), *RASSF1A* (76% and 29%) and *RAR-β*, (40% and 8%, ns), and were more likely than EA women to have 3 or more methylated genes (80% vs 0%, p < 0.005). No differences in methylation patterns were evident between AA and EA women with ER+/PR+ tumors, or among older women (> 50 years). More recently, Wang and colleagues [9] used pyrosequencing to examine gene-specific (*p*16, *RASSF1A, RARβ2, ESR1, LINE1, CDH13, HIN1*, and *SFRP1)* methylation in breast tumor DNA from 32 AA and 33 EA breast cancer patients. However, this study did not replicate the gene-specific findings of Mehrotra, et al, only noting racial differences in methylation for *CDH13*, and not in *HIN-1* and *RASSF1A.* In their analysis, they did observe that the greatest differences in methylation of *CDH13* were among women with ER negative breast cancer and among those younger than 50 years.

**Figure 1:**
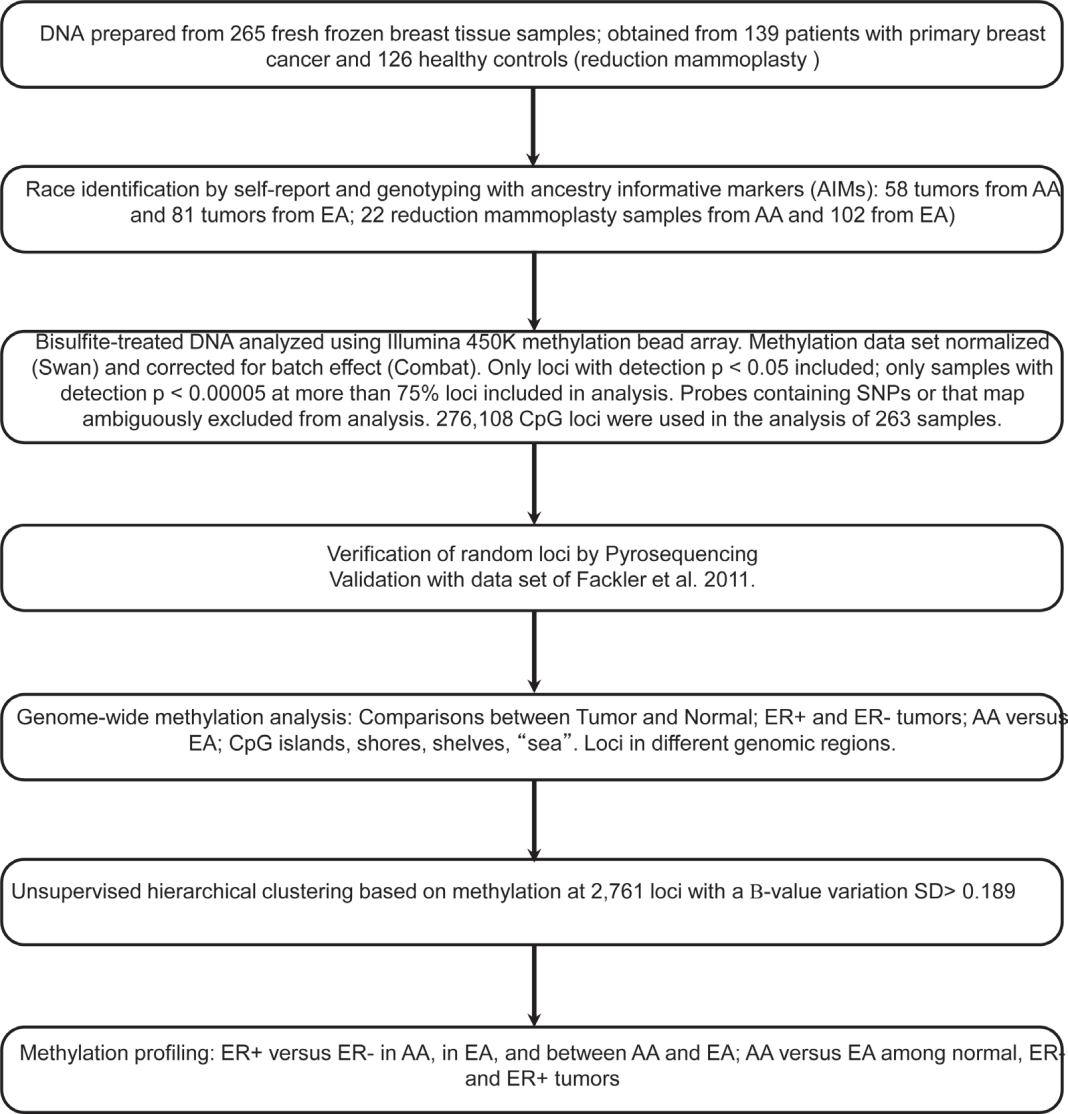
Schema of study design and data analysis plan for DNA methylation profling in relation to breast cancer among AA and EA women

Studies interrogating large numbers of loci have indicated that, in addition to racial differences, methylation patterns also differ according to breast cancer subtypes [9,10,11,12]. These results are provocative and provide a hint that the molecular basis of aggressive breast cancer may be related to gene methylation, and that methylation patterns for aggressive tumors may differ by ancestry. With recent capabilities to examine genome-wide differential patterns of methylation, we sought to determine if methylation patterns distinguish tumors by race and by ER status, and if there are differences between AAs and EAs within ER subgroups. Using the Illumina Infnium HumanMethylation450 BeadChip (from here on called 450K), we evaluated DNA methylation in breast tumor tissue from AA and EA women with cancer and in breast tissue from AA and EA women without cancer who were undergoing surgical reduction mammoplasty, as illustrated in Figure 1.

## RESULTS

Table 1 shows the clinical characteristics of the patients from whom tumor DNA was derived. There was a higher frequency of ER negative tumors among AA women (45%) than EAs (31%). Genotyping for a panel of 24 Ancestry Informative Markers (AIMs) in the total cohort verifed self-reported race for all samples except for two from reduction mammoplasty patients (data not shown). These patients who self-reported as AA but had less than 15% African ancestry according to the AIMs were excluded from the analyses. We also excluded an additional patient for whom ER status was not available. A total of 262 DNA samples (58 and 80 tumor samples from AA and EA women, respectively, and 22 and 102 normal breast samples from AA and EA women) were available for analysis for CpG methylation levels using the 450K BeadChip.

**Table 1:** Characteristics of patients with primary breast cancer from whom tumor tissue was derived

	African-American (n=58) (%)	European American (n=80) (%)
Age		
<49	18 (32)	22 (28)
50-68	20 (34)	29 (36)
>69	20 (34)	29 (36)
		
Estrogen Receptor Status		
Negative	26 (45)	25 (31)
Positive	32 (55)	55 (69)
		
Progesterone Status		
Negative	31 (53)	41 (51)
Positive	27 (47)	39 (49)
		
HER2 Status *§	N=54	N=74
Strong	7 (13)	26 (35)
Weak	1 (<1)	4 (6)
Negative	47 (87)	44 (59)
		
Histological Grade**	N=57	N=78
I (well differentiated)	0	2 (3)
II (moderately differentiated)	13 (23)	8 (10)
III (poorly differentiated)	44 (77)	68 (87)
		
Stage		
in situ	1 (1)	0
I	10 (18)	8 (9)
IIA	16 (28)	27 (35)
IIB	15 (26)	21 (27)
IIIA	7 (12)	14 (18)
IIIB	1 (1)	4 (5)
IIIC	5 (9)	1 (<1)
IV	3 (5)	5 (6)

### Validation of Illumina Infnium Human Methylation 450 Bead Chip results

**Figure 2:**
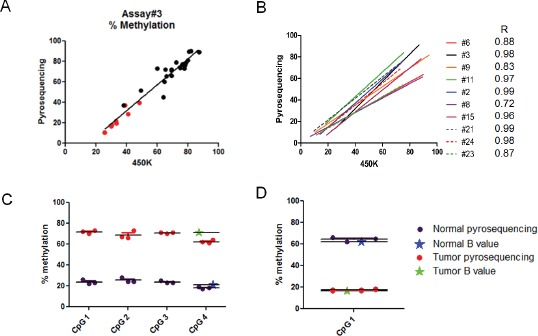
Verification of Infnium 450K results by pyrosequencing Twenty (20) tumor and 8 reduction mammoplasty samples were analyzed using pyrosequencing assays designed at 10 randomly chosen 450K CpG loci (Supplementary Table 1). The percent methylation determined by pyrosequencing was plotted against the β-value determined by Infinium 450K analysis multiplied by 100. A. Panel shows representative results (assay #3) for each of the tumor (black dots) and reduction mammoplasty (red dots) samples. B. Panel shows the correlation plots for each of the 10 assays with the Pearson's correlation indicated for each. Assays #6, #8, #9, and #15 were subsequently shown to map ambiguously (see M&M). C & D. Representative results comparing the β-value determined by 450K analysis at a single CpG locus (CpG4) with the methylation levels determined in the same two samples by pyrosequencing (points are triplicate assays). Note that adjacent (<200 bp in these assays) CpG dinucleotides show similar levels of methylation as the single CpG locus interrogated by the 450K probe. Panel C shows a locus that is hypermethylated in tumors compared to normal breast tissue. Panel D shows a locus that is hypomethylated in tumors compared to normal breast tissue.

We used two approaches to obtain both internal and external validity of our findings of differential methylation between AAs and EAs according to ER status. For internal validation, e.g., to assess the accuracy of the methylation levels determined by the 450K BeadChip analysis, pyrosequencing assays were developed that encompassed ten differentially methylated CpG loci interrogated on the 450K Bead Chip. Following analysis of 20 tumor samples and 8 reduction mammoplasty samples, a high degree of correlation was observed between methylation levels determined by the two independent methods (Fig. 2A & B). Furthermore, pyrosequencing showed that, in the majority of cases, the methylation levels determined at a single CpG locus refected the methylation levels of nearby CpG dinucleotides not assessed by 450K probes (Fig. 2C & D). Together, these results provide confidence in the 450K Bead Chip approach to accurately measure methylation levels over at least small genomic regions (e.g. promoter regions) using only isolated probes.

We also used the results from Fackler et al. [12] as another source population for external validation of our results. They identified a list of 40 CpG loci that were differentially methylated with respect to ER status, with 27 hyper-methylated in ER-positive tumors, and 13 hyper-methylated in ER-negative tumors. Thirty-five of those 40 probes were also interrogated in the 450k platform used herein, with 18 of them included in the final dataset. We found that the patterns of hyper- and/or hypo-methylation for all of these 18 probes were consistent between our study and the one by Fackler et al. (Supplemental Table 1; Pearson's correlation coefficient = 0.9612) thus providing external validation of our results in terms of detecting methylation changes by ER status.

### Genome-wide methylation levels

**Figure 3:**
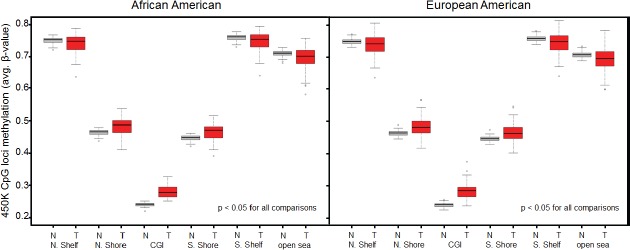
Genome-wide differences in methylation levels between tumors and reduction mammoplasty samples stratified by location of interrogated CpG locus Average methylation levels at loci within CGIs and CGI-shores were consistently higher in tumors compared to normal controls regardless of race (Welch's t-test). Average methylation levels at loci outside of CGIs (i.e. CGI-shelves and open sea) were consistently lower in tumors compared to normal controls regardless of race (Welch's t-test). N, normal (reduction mammoplasty); T, tumor; N.Shelf, “North Shelf”; N. Shore, “North Shore”; S. Shore, “South Shore”; S. Shelf, “South Shelf”.

**Figure 4:**
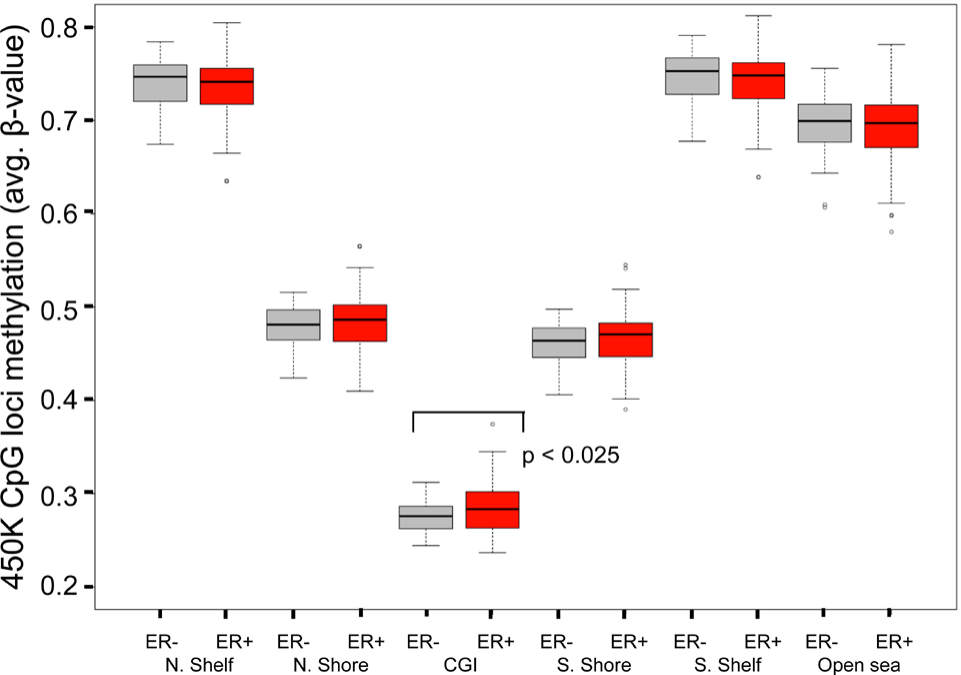
Genome-wide differences in methylation levels between ER + and ER- tumors stratified by location of interrogated CpG locus Average methylation levels at loci within CGIs were higher in ER+ tumors compared to ER- tumors (p < 0.025, Welch's t-test). All other differences were not statistically significant. N, normal (reduction mammoplasty); T, tumor; N.Shelf, “North Shelf”; N. Shore, “North Shore”; S. Shore, “South Shore”; S. Shelf, “South Shelf”.

As described in Materials and Methods, we excluded probes that contained SNPs or were shown to map ambiguously, leaving a total of 276,108 CpG loci in the data set. Comparisons of genome-wide methylation levels using these loci were first made between tumor and normal breast tissue. In aggregate, methylation levels at all loci were significantly higher in breast cancer samples than in normal breast tissue from women undergoing breast reduction surgery (Supplemental Fig.1). This trend continued when comparisons were made across AAs and EAs, but was not statistically significant for the AA samples, possibly due to the smaller number of reduction mammoplasty samples from AA women (Supplemental Fig.1). As anticipated, genome-wide differences in methylation levels were more pronounced when CpG loci were stratified with respect to their location relative to CGIs. Indeed, methylation levels at CpG loci in both CGI and CGI-shores (regions up to 2kb distant from CGI) were significantly higher (Welch's t-test, p < 0.05) in tumor samples compared to normal samples, regardless of race (Fig. 3). Furthermore, consistent with other studies showing hypo-methylation in tumors outside of CGI, methylation levels at CpG loci in CGI-shelves (2-4kb from CGI) as well as “open sea” loci (isolated CpGs) were lower in tumor samples compared to reduction mammoplasty samples from both AA and EA patients. When comparing all tumors by ER status, it appeared that genome-wide methylation levels at CGIs were higher in ER positive tumors than in ER-negative tumors (Fig. 4). Although the trend was similar when stratifying by AA or EA, associations were no longer significant (Supplemental Fig.2), and no significant differences in genome-wide methylation levels were observed in ER-negative and ER-positive tumors of different ancestry (Supplemental Fig. 3). In reduction mammoplasty samples, no significant differences in overall methylation levels were observed in DNA from women of AA or EA ancestry.

### Clustering analysis

**Figure 5:**
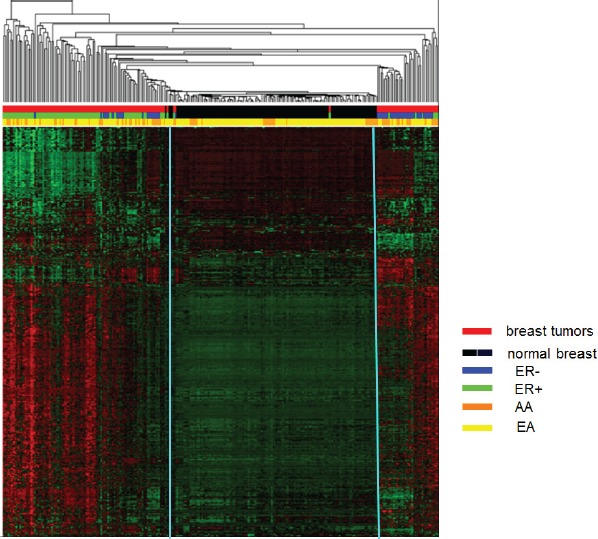
Unsupervised hierarchical cluster analysis of the most varied CpG loci probes among tumor and normal breast tissues (2,761 probes, SD > 0.189) Based on the average linkage and Manhattan distance metric on 139 tumors and 126 controls, three distinct clusters were identified. Cluster 1 is primarily tumor samples (red bars) enriched for ER-positive tumors (green bars); cluster 2 is predominately normal samples (black bars); cluster 3 is primarily tumor samples (red bars) enriched for ER-negative tumors (blue bars). Status: red=tumor and black=normal; ER: green=ER-positive and blue=ER-negative; Race: orange=AA and yellow=EA. In heat map, red lines indicate hypermethylation and green lines indicate hypomethylation.

Unsupervised hierarchical clustering based on the average linkage and Manhattan distance metric was employed to analyze 2,761 probes that showed the most variable DNA methylation levels (SD > 0.189) across the breast tumor panel. As show in Figure 5, three major clusters emerged (from left to right). The first cluster is enriched for ER-positive (green) tumors (red); the second cluster is predominately normal samples (black); the third cluster is enriched for ER-negative (blue) tumors (red).

The methylation patterns clearly distinguish the control tissue samples from breast cancer samples, as well as ER-positive from ER-negative tumor samples. The strongest classification was between normal (black) and tumor (red), and then between ER-positive (green) and negative (blue) tumors. Clustering analysis revealed some degree of ancestry delineation in normal tissue, with little delineation among cancer patients. This is in contrast to the genome-wide methylation analysis described above and shown in (Supplemental Fig. 2), where there were no significant differences in *overall* levels by race and ER status.

### Differential methylation by ancestry and ER status

**Figure 6:**
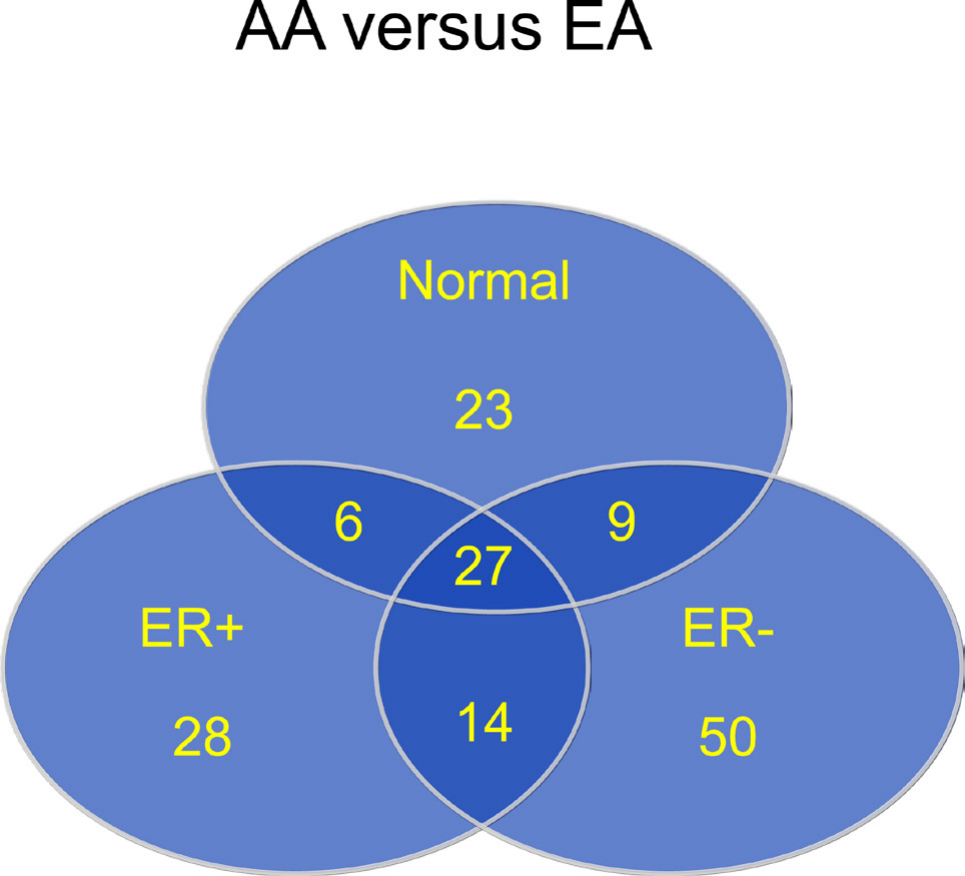
Most differentially methylated CpG loci by race (AA versus EA) in normal and tumor tissue, and by ER status

The Wilcoxon rank-sum test was used to evaluate the difference in DNA methylation β values for each probe in each of the comparisons made. As described in the Methods, CpG loci differentially methylated between groups were defined as those with a mean β value difference (|delta β|) of at least 0.17. Corrections for multiple testing were performed using the Benjamini and Hochberg approach [13]. Figure 6 shows the distribution of 157 CpG loci whose methylation levels varied by ancestry, ER status, or within ancestry/ER groups, with comparisons identifying differentially methylated regions between 3 groups: 1) AA and EA in normal tissue, 2) AA vs EA in ER positive tumors, and 3) AA vs EA in ER negative tumors. The distributions of the top 78 differentially methylated CpG loci between tumors from AA and EA women according to ER status. There were almost twice as many differentially methylated loci in ER-negative than in ER-positive tumors, with equal numbers of hyper- and hypo-methylated loci.

The 20 loci most differentially methylated by race are shown in Supplementary Table 2. Among the top 20 differentially methylated loci by ancestry in ER-negative tumors, 16 loci were located in known gene regions, and a total of 12 out of 16 of those loci were located in genes that either encode transmembrane proteins (TMEM57, ACPT, XKR6, FAM176A, CDH4) and extracellular matrix proteins (FMOD and C6orf186), or are associated with inflammatory responses (FAM19A5, THRSP, CERK, NLRP6). When examined collectively, the related accession numbers of the top differentially methylated loci showed distinct phenotype patterns. For example, loci differentiating ER-negative tumors have also been shown to be significantly associated with breast cancer, lipid levels, cardiovascular disease, bone density, osteoporosis and arthritis. The only phenotype associated with loci differentiating normal AA and EA tumors were found to be associated with diabetes.

## DISCUSSION

In genome-wide DNA methylation analysis of breast tumors from AA and EA women, and breast tissue from women without cancer undergoing surgical reduction mammoplasty, we found numerous differences. Tumor tissue was characterized by hyper-methylation at CpG loci in CGI and CGI-shores, and hypo-methylation at loci located in CGI-shelves and “open sea”. Hierarchical clustering provided partial differentiation by ancestry in non-cancer tissues, but this delineation was not seen in breast tumors. In addition, clustering of breast tumor methylation patterns could, to a degree, distinguish ER status. In examination of tumors from AA and EA women by ER status, there were many more loci differentially methylated by race in ER negative than in ER positive breast cancers.

This is the first study to apply a genome-wide approach to investigate associations between DNA methylation and breast cancer disparities, and to examine differences by ancestry within ER groups. Our findings of greater methylation differences between EAs and AAs within ER-negative tumors are consistent with the suggestive earlier findings showing that, for a panel of five candidate genes, *HIN-1* (*SCGB3A1*), *Twist (TWIST1), Cyclin D2 (CCND1)*, *RASSF1A, RARB*, there were differences by ancestry. These differences were only apparent within tumors that were ER-negative and from women diagnosed before age 50 years, where greater methylation was observed for AA than for EA women [8]. Similarly, in a candidate gene study by Wang et al [9], differential methylation between tumors from AAs and EA women were only observed for ER-negative tumors. It is interesting to note that all 36 genes that were differentially methylated by race among ER-negative tumors in our analysis are novel, and do not include the candidate genes that showed differential methylation by race and ER status in these previous studies. These differences in findings could be related to methodological approaches, but may also be similar in concept to epidemiological studies, wherein findings regarding polymorphisms in candidate genes are not replicated in genome-wide association studies (GWAS). Similar to a GWAs study, our genome-wide analysis revealed differential methylation by race in ER negative tumors in genes that had not been previously hypothesized and studied in candidate gene approaches, similar to the consistent findings between a variant in 8q24 and risk of breast cancer. These findings illustrate the power of taking an agnostic approach to identify the genes that best define differential etiologic pathways in aggressive breast cancers by ancestry.

We included DNA from normal breast tissue from women undergoing reduction mammoplasty so that we could have a representation of ‘normal’ methylation differences between AAs and EAs, which has been observed in newborn cord blood as well as within normal prostate tissue and leukocytes from healthy women. We then removed those loci that were differentially methylated in the reduction mammoplasty samples from our analysis when examining differences by ancestry and ER status. This approach provides more assurance that the loci that are differentially methylated are related to cancer, and not just normal differences between EAs and AAs. The loci that were most differentially methylated in normal breast tissue between EA and AA women were not comparable to those in earlier studies of cord blood and of leukocytes. In the study of DNA methylation in leukocytes [4], the methodology ([3H]-methylation acceptance assay) assesses methylation at all genomic CpG dinucleotides, including those in repetitive sequences, while the 450K platform does not. We did compare our results from normal breast tissue to those most differentially methylated by race in cord blood from newborns [5], obtained using the Illumina 27K. Among the 4216 loci that were significant (p<0.01) in that study and were also included in our filtered dataset, only 189 were also significant (p<0.01) in our study. This is not surprising, since it has now been established that DNA methylation patterns between children and adults are remarkably different [14,15,16]. Furthermore, Zhang et al recently showed that DNA methylation profiles are different according to tissue type, with notable differences between breast tissue and blood from the same patients [17].

In the hierarchical clustering, we observed some distinct separation between EA and AA DNAs for normal tissue, but not in cancer tissues. Rather, there was greater separation in cases by ER status than by race, suggesting that ER status is a better distinguisher of tumor types than race. Although we were able to determine that racial differences in methylation were greatest in women with ER negative breast cancers, disentangling the relationships between DNA methylation, race and tumor aggressiveness (characterized as ER-negative disease in this analysis) will require analysis in a much larger sample set to evaluate the independent effects of ancestry and tumor characteristics on DNA methylation patterns.

Until recently, candidate gene approaches were taken to investigate the role of DNA methylation in carcinogenesis, focusing on a defined set of targeted genes, many of which were identified based on mutation patterns in tumors. These genes were often tumor suppressor genes, and affected cell growth control, migratory capability, evasion of immune surveillance, and promotion of angiogenesis. For breast cancer, methylation has been noted in genes including BRCA1, p16, E-cadherin, H-cadherin, ATM, CST6, cyclin D2, PTEN, RASSF1A, APC, RARb2, GSTP1, ER, PR, as well as numerous other genes related to multiple pathways relevant for carcinogenesis [8,9,18]. As noted above, a candidate gene approach was also taken by Mehrotra and colleagues to investigate associations between methylation patterns, race, and ER subgroups of breast tumors [8]. In the last few years, additional technology has become available to examine the genome in a more agnostic approach, by scanning loci across the human genome for methylation patterns, evaluating the role of methylation in differentiating tumor characteristics and survival outcomes. For example, Kamalakaran and colleagues [11] used a Methylation Oligonucleotide Microarray Analysis (MOMA) to analyze thousands of genomic loci including most CGIs, comparing DNAs from 108 breast tumors and 11 normal adjacent tissues. They used hierarchical modeling to examine clustering in relation to breast cancer subtypes, and found that clusters separated into 3 groups, one primarily luminal A and another basal-like (negative for ER, PR and HER, and over-expression of EGFR and CK5/6), with a third non-specific group that included normal samples, similar to our findings of separation between normal and tumor and by ER status. The genes identified in the Kamalakaran study that were differentially methylated between subgroups (luminal A and basal-like) are not the same genes for which expression arrays first identified the intrinsic subtypes [19]. This lack of association between subtype clustering by methylation and by gene expression implies that methylation does not, in all cases, result in the predicted effects on gene expression. Holm et al. [10] also used an array based approach (Illumina Golden Gate) to examine methylation patterns of 1505 loci in 807 pre-selected cancer-related genes in relation to breast cancer subtypes in a sample of tumors from 189 women with breast cancer. In hierarchical clustering of the 332 most variably methylated loci, samples clustered primarily according to ER status, with further division of the ER-positive tumors into luminal A and another group containing a mixture of subtypes. Cluster affiliation also separated out survival outcomes and S-phase fractions. The same Illumina Golden Gate platform was also used by Christensen and colleagues [20] to examine methylation profiles in breast tumors from 165 women. Using recursively partitioned mixture methodology, predictive factors for class membership included tumor size and race, although the number of minorities in the sample was small (13 AA, 10 Hispanic).

Fackler et al used the Illumina Infinium HumanMethylation 27K array to query methylation loci across the genome in breast cancer [12]. In that study consisting of DNAs from 103 women with breast cancer, there were more hyper-methylated loci in ER-positive than ER negative tumors. In line with this observation, we found that methylation levels at CGIs were higher in ER positive tumors than in ER-negative tumors. Because our study included both AA and EA women with breast cancer, we were able to further analyze data stratifying by race; in this race-specific analysis, the trend for higher methylation levels in ER-positive tumors remained, but the association was not statistically significant. In our analysis, there were 28 and 50 loci that were differentially methylated by ancestry in ER-positive and ER-negative tumors, respectively, corresponding to 15 and 36 genes. The greater number of differentially methylated genes by ancestry in ER-negative tumors may refect the fact that ER-negative breast cancers are more biologically diverse and comprised of more breast cancer subtypes [21,22].

The genes most differentially methylated between AAs and EAs within ER-negative and ER-positive tumors were distributed sporadically in signaling networks, with no significant enrichment for any pathway in either distinct group, suggesting that there are no dominant signaling pathways underlying racial disparities in breast cancer. As noted above, the top 20 differentially methylated loci by ancestry in ER-negative tumors were located in genes that either encode transmembrane proteins and extracellular matrix components or are associated with infammatory responses. This suggests that tumor-microenvironment interactions in ER-negative tumors may behave differently between AA and EA patients. In ER-positive tumors, only 11 loci were located in known gene regions and were distributed diversely without apparent targets or mechanisms. The fact that a large proportion of the top differentially methylated loci were not in the transcriptional regions where CpG islands or promoter regions reside, but rather in gene bodies or shores, highlight the growing understanding of the importance of DNA methylation in these regions for transcription regulation and tumor initiation [23,24].

This is the first molecular epidemiological study to address the role of DNA methylation in racial disparities in breast cancer, and to examine genome-wide methylation differences according to ER status and race. Importantly, the results are consistent with limited previous candidate gene approaches and gene expression studies, showing that ER-negative tumors appear to show complex differences by race, with β values and p values showing distinct differences in methylation between and across race and breast cancer subtypes. Although results need to be replicated in a larger study, this epidemiologic observational study is the first step and lays the foundation to follow-up with laboratory-based gene-by-gene functional studies to examine DNA methylation in greater depth.

In summary, we found that genome-wide methylation patterns differ by ER status, and importantly, that there are substantially more loci differentially methylated between AAs and EAs among women with ER-negative breast cancer. These findings suggest that there may be distinct differences in the etiology of aggressive breast cancer by ancestry; in-depth investigation of the genes that are most differentially methylated by ancestry in ER-negative breast cancer may provide better insight into etiology and prevention, with potential implications for therapeutic approaches to specifically target ER-negative breast cancer in AA women.

## MATERIALS AND METHODS

### Tissue Samples

The overall study design and analysis are illustrated in Figure 1. We initially evaluated DNAs from 265 women, in total. DNA derived from fresh frozen breast tumor tissue from 58 AA and 80 EA women was obtained from the Pathology Resource Network (PRN) at Roswell Park Cancer Institute (RPCI). Breast tissue specimens are routinely collected from all surgeries, after patient consent for use of remnant tissue for research, snap frozen, and stored at -80 degrees C. Genomic DNA was isolated from banked specimens using the Puregene (Gentra D70KA) DNA purification protocol, as per manufacturer's instructions, and linked with clinical information by the Clinical Data Network at RPCI. DNA from normal breast tissue was available from 22 AA and 102 EA women undergoing reduction mammoplasty. Surgically removed tissue was inspected and determined to be free from gross pathologic abnormalities, as previously described [6]. Epithelial tissues were blunt dissected and snap frozen in liquid nitrogen, and DNA was extracted using a MasterPure DNA purification kit (Epicentre). To validate self-reported ancestry, we genotyped breast tumor and normal DNA samples using Sequenom technology with 24 AIMs shown to be precise in estimating European admixture in AA populations, and verified ancestry using the STRUCTURE program [25].

### DNA Methylation Analysis

Genome-wide methylation analysis was carried out using the Illumina Infnium HumanMethylation450 BeadChip platform, an oligonucleotide array that interrogates > 485,000 CpG dinucleotides per sample at single-nucleotide resolution (http://www.illumina.com/products/methylation_450_beadchip_kits.ilmn). The BeadChip covers 99% of RefSeq genes, with an average of 17 CpG sites per gene region distributed across the promoter, 5'UTR, first exon, gene body, and 3'UTR. The chip covers 96% of CpG islands (CGIs), with additional coverage in CGI shores and the regions flanking them (CGI shelves), as well as in so-called “open sea” regions [26,27]. In order to minimize the impact of batch effects, DNA samples from tumors were randomized on plates according to age, ancestry, and ER status and interspersed with samples from the normal tissues randomized by age and ancestry [28]. Following bisulfite treatment of DNA using the Zymo EZ DNA methylation kit, subsequent steps for the HumanMethylation450 BeadChip analysis were carried out according to the manufacturer's instructions. BeadChips were scanned using the Illumina BeadArray Reader with High-Density (HD) Technology and BeadScan software.

### Statistical Analysis

The raw intensity of Illumina Infnium HumanMethylation450 BeadChip was scanned and extracted using BeadScan in GenomeStudio module. The bead information was summarized into BeadStudio IDAT files and then processed by the R package minfi. The resultant methylation levels (β value) ranged between (0, 1), with 0 absent methylation and 1 complete methylation. The SWAN normalization procedure was performed to correct for design bias [29] and the ComBat algorithm was performed to correct batch effects [30,31].

### Sample and probe/locus quality control

Rigorous quality-control criteria were used for filtering at both the locus and sample levels [32]. Only loci with a median [33] detection p value < 0.05 were retained for analysis, and only samples with detection p values < 1×10-5 at more than 75% of CpG loci passed the performance criteria for inclusion. Probes recently shown to contain SNPs and/or those that were ambiguously mapped [33,34,35] were excluded from the analysis. As shown in Figure 1, the final dataset contained 276,108 CpG loci (hereafter referred to simply as “CpG loci”) across 263 samples.

### Genome-wide statistical analysis

Genome-wide methylation levels (defined as average methylation β-values of all interrogated CpG loci of each sample) were compared between tumor and normal tissue, stratified by ancestry (EA vs AA) and by ER status (positive vs negative). The analyses were further stratified by separately examining CpG loci within CGIs, CGI shores, CGI shelves and “open sea”. We then performed unsupervised hierarchical clustering on probes that showed the most variable DNA methylation levels across the breast tumor panel (2761 probes, SD>0.189). Manhattan distance and average linkage were employed in clustering analysis.

### Locus-specific statistical analysis

Differences in DNA methylation β-values for each probe were evaluated between AAs and EAs in DNAs from normal tissues and from tumors, stratified by ER status, as well as comparisons of ER positive vs ER negative in each of the two racial groups separately. CpG loci differentially methylated between selected groups of interest were defined as those with a mean β-value difference (|delta β|) of at least 0.17, as recommended by Illumina [36,37]. This Wilcoxon rank-sum test was used to evaluate the statistical significance for each probe in each of the comparisons made. Multiple testing corrections were performed using the Benjamini and Hochberg approach with significantly differential methylation defined at FDR-adjusted p < 0.05.

### Validation of differentially methylated loci

We validated our findings using both pyrosequencing and *in silico* confirmation. Independent DNA aliquots from 20 of the fresh frozen breast tumors and 8 samples from reduction mammoplasty used in the 450K analysis were bisulfite-treated and used to perform pyrosequencing to follow up on 10 randomly selected loci that were shown to be either hyper-methylated or hypo-methylated in tumors compared to normal breast tissue by Illumina Infinium 450K methylation analysis. Pyrosequencing assays for these loci were kindly provided by Gerald Schock at Qiagen (see Supplemental Table 3 for location of loci and pyrosequencing primers). We next compared our results to those from a study [12] in which the Illumina Infinium 27K platform was used to examine differential methylation by ER status among EA women, finding 27 loci that were hypermethylated in ER positive tumors and 13 that were hypermethylated in ER negative tumors. The 450K platform contains 35 of these 40 loci, with 18 of them contained in our final dataset.

## Supplementary Figures and Tables




