# Supplementary material for: Genome-wide methylation patterns provide insight into differences in breast tumor biology between American women of African and European ancestry
Source: Oncotarget. 2013 Nov 29;5(1):237–48. doi: 10.18632/oncotarget.1599 (PMC3960204; doi:10.18632/oncotarget.1599)
Supplement: Supplementary file 1 [file oncotarget-05-0237-s001.pdf]

Genome-wide methylation patterns provide insight into differences in breast tumor biology between American women of African and European ancestry - Ambrosone et al

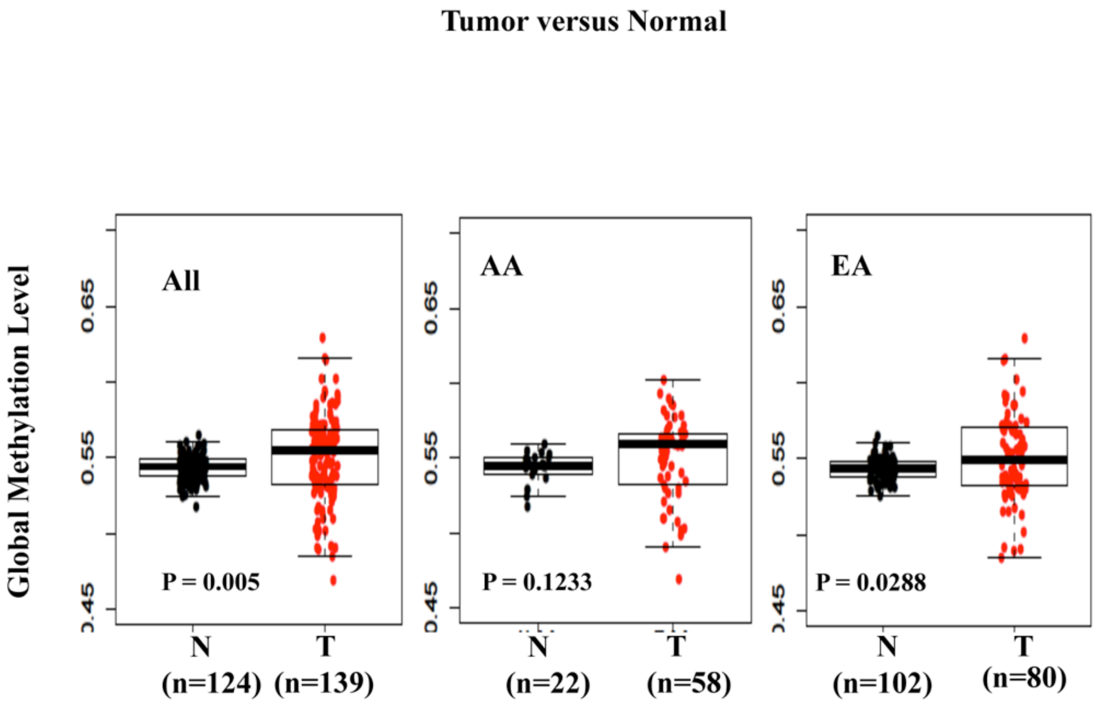

Figure S1: Genome-wide differences in methylation levels between tumors and reduction mammoplasty samples. On average, methylation levels at all CpG loci were consistently higher in tumors compared to normal controls (Welch's t-test). This was the case when stratified by ancestry, but the difference between normal and tumor was only statistically significant in EA women.

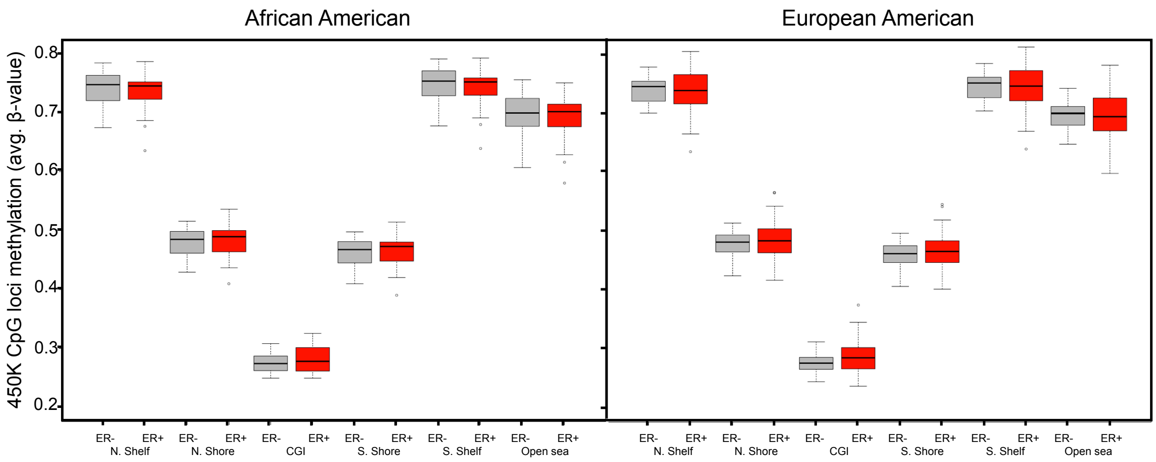

Figure S2: Genome-wide differences in methylation levels between ER + and ER- tumors stratified by ancestry and location of CpG loci with respect to CGIs. Although average methylation levels at loci within CGIs and CGI-shores were higher in ER+ tumors compared to ER-tumors regardless of ancestry, differences were not statistically significant (Welch's t-test). N.Shelf, "North Shelf"; N. Shore, "North Shore"; S. Shore, "South Shore"; S. Shelf, "South Shelf".

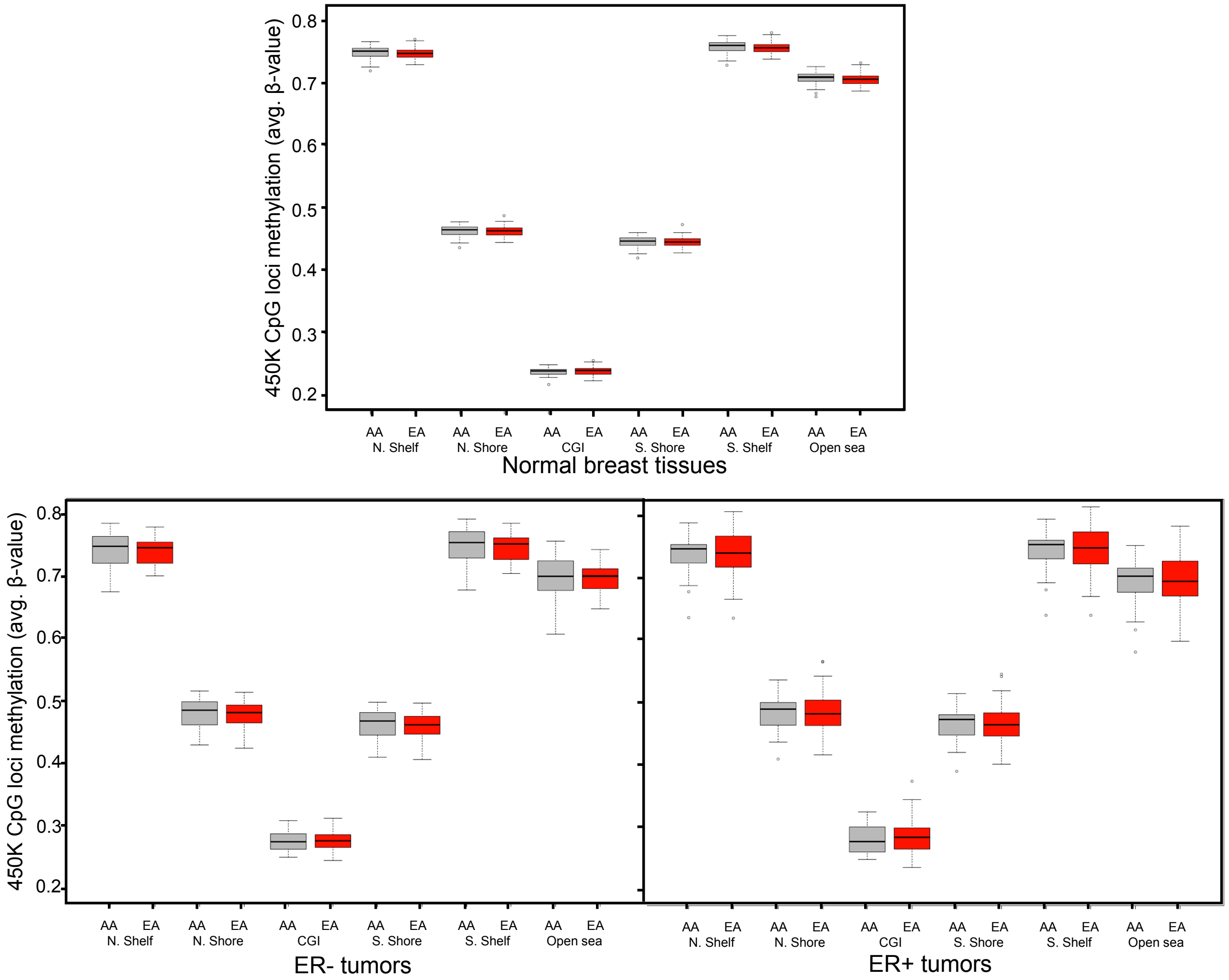

Figure S3: Genome-wide differences in methylation levels between EA and AA women and location of CpG loci with respect to CGIs in normal tissue, and within tumors stratified by ER receptor status.
